# Normative scores for a brief neuropsychological battery for the detection of HIV-associated neurocognitive disorder (HAND) among South Africans

**DOI:** 10.1186/1756-0500-3-28

**Published:** 2010-01-29

**Authors:** Dinesh Singh, John A Joska, Karl Goodkin, Enrique Lopez, Landon Myer, Robert H Paul, Sally John, Henry Sunpath

**Affiliations:** 1 Department of Psychiatry, University of Kwa-Zulu Natal, Durban, South Africa; 2 University of Cape Town, Observatory, South Africa; 3 Department of Psychiatry and Behavioral Neurosciences of Cedars-Sinai Medical Center, Los Angeles, USA; 4 Department of Psychiatry and Biobehavioral Sciences, University of California, Los Angeles, USA; 5 Mailman School of Public Health, Columbia University, USA; 6 Department of Psychology, University of Missouri, St Louis, USA; 7 McCord Hospital, Durban, South Africa

## Abstract

**Background:**

There is an urgent need to more accurately diagnose HIV-associated neurocognitive disorder (HAND) in Africa. Rapid screening tests for HIV-associated dementia are of limited utility due to variable sensitivity and specificity. The use of selected neuropsychological tests is more appropriate, but norms for HIV seronegative people are not readily available for sub-Saharan African populations. We sought to derive normative scores for two commonly used neuropsychological tests that generate four test scores -- namely the Trail-Making Test (Parts A and B) and the Digit Span Test [Forward (DSF) and Backward (DSB)]. To assess memory and recall, we used the memory item of the International HIV Dementia Scale (IHDS).

**Findings:**

One hundred and ten HIV seronegative participants were assessed at McCord Hospital, Durban, South Africa between March 3^rd ^and October 31^st^, 2008. We excluded people with major depressive disorder, substance use abuse and dependence and head injuries (with or without loss of consciousness). All the participants in this study were African and predominantly female with an average age of 28.5 years and 10 years of education. Age and gender influenced neuropsychological functioning, with older people performing worse. The effect of gender was not uniform across all the tests.

**Conclusion:**

These two neuropsychological tests can be administered with the IHDS in busy antiretroviral clinics. Their performance can be measured against these norms to more accurately diagnose the spectrum and progression of HAND.

## Introduction

Infection of the central nervous system by the human immunodeficiency virus (HIV) is frequently associated with potentially debilitating forms of neurocognitive disorders. HIV-associated dementia (HAD) occurs in approximately 10-15% of all individuals with HIV/AIDS and is more common in late stages of infection[1]. The less severe disorder (mild neurocognitive disorder (MND) and sub-clinical neurocognitive impairment more common, occurring in 30-60% of people infected with HIV depending on disease stage [2,3]. Collectively, these disorders are referred to as HIV-associated neurocognitive disorders (HAND) [4].

While neuropsychological (NP) test batteries are regarded as the gold standard by which to diagnose HAND, particularly HAD [5-8], these are not always available or appropriate in busy clinical settings. It is important to detect HAD because of its severity despite a greater than 40% decline in the incidence of HAD [2,9] since the widespread introduction of highly active anti-retroviral therapy (HAART). Without antiretroviral (ARV) therapy, death may ensue within 6 months [10]. South Africa and other sub-Saharan countries follow the WHO guidelines for initiating ARVs in resource constrained settings. People with HAD are eligible to HAART, regardless of CD4 cell count. In addition to the importance of diagnosing HAD and initiating HAART on this context, there is growing evidence that MND and sub-clinical neurocognitive impairment should also be diagnosed and treated[2].

Many screening tests have been used to detect HAD, such as the HIV dementia scale (HDS) [6], the EXIT interview [11], mental alternation tests [12], the modified Memorial Sloan-Kettering scale [13], and the International HIV Dementia Scale [8]. These tools may have limited clinical utility because they are insensitive to the milder end of the spectrum of HIV-associated neurocognitive impairment [14]. Longer NP batteries require local normative data for comparison. Ultimately, a balance needs to be found between longer but less practical batteries, and shorter, but less sensitive ones.

The American Academy of Neurology research nomenclature and diagnostic process for HAND have recently been updated [4]. This approach not only specifies the domains most affected by HIV, namely speed of processing information, memory, attention/concentration, learning, executive functioning, motor and psychomotor speed but also requires that specialised NP test batteries are employed. The administration of detailed batteries tends to be restricted to research settings, and requires trained neuropsychologists. In order to address the gap between longer NP batteries and the brief screening tools, selected NP tests, which represent the domains most affected by HIV infection, need to be identified for use in international settings. In addition, these tests need to be measured against normative data obtained from HIV seronegative people in the population in question. For example, there is some concern that "speed" in completing cognitive tests is not emphasized equally across regions of the world. If true, healthy controls outside of US and Europe would perform more poorly on tests of neurocognitive or motor speed when compared to normative data obtained in the US or Europe, where most neurocognitive tests have been developed [15].

Published norms for neurocognitive tests cannot be used in all populations. For example, a comparison of normative scores for the Trail Making Test from various countries and cultures showed that scores are not equivalent and can lead to serious diagnostic errors[16]. Relevant to the current study, population normative scores are not widely available for Africans [7]. Three quarters of all people living with AIDS live in sub-Saharan Africa [17]. South Africa is the country with the highest number of HIV infections worldwide and is a region where HIV-1 clade C is predominant. While it has been proposed that clade C was less neurovirulent than other, better studied clades (like B), it has recently been suggested that neurocognitive impairment occurs at similar rates. There are no guidelines to assess HAND in Africa; yet, the importance is very high from both clinical and research perspectives as both rely on normative data to generate clinical classifications of impairment. The purpose of this study was to establish population normative scores for a select number of NP tests that can be used in South African antiretroviral clinics to assess the neurocognitive domains most commonly affected by HIV infection.

## Methods

### Sample and setting

This study was conducted at McCord Hospital, Durban, South Africa between March 3^rd ^and October 31^st^, 2008. McCord Hospital has a voluntary counselling and testing clinic (VCT) for HIV and an ARV clinic. At the VCT clinic from January through December, 2008, a total of 1,052 patients received voluntary counselling and testing. HIV testing is free and confidential and is available to anyone requesting a test.

Participants were recruited from the VCT clinic. To limit selection bias, we recruited participants on different days and selected people from the list of all people registered for the day using a random numbers list. We excluded people who were: HIV seropositive, under 18 years of age, who reported a history of any substance abuse or dependence in the past three months (including alcohol), those who reported any past psychiatric history, or any history of conditions that would affect neurocognitive function e.g. past head injury (regardless of loss of consciousness), cerebrovascular accidents, or seizure disorders. Primary languages of the participants were either *isi*Zulu or English. After determining eligibility criteria, one hundred and ten consenting participants were assessed in a private room to minimize distractions from noise and other operational distractions in the VCT clinic. This study was reviewed and approved by the hospital's research and ethics committee prior to study initiation.

### Measures

#### NP Test battery

We chose individual tests which were sensitive to domains affected by HIV infection, could be administered by non-psychologists without specialized equipment, and would require no longer than 20 minutes. Five tests were initially selected; each of the tests selected have been successfully administered before on HIV seropositive people but not in South Africa [5,8,18-20].

*Attention and concentration *was assessed using Digit Span Forward (DSF) and Digit Span Backwards (DSB). Digit span tests are frequently used because they are sensitive measures of both attention and working memory[16]. *Speed of information processing *was assessed with the Trail Making Test Part A (TMT A), and *Executive Function *was assessed by Trail Making Test Part B (TMT B). The latter two tests also measure psychomotor functions. While the Grooved Pegboard Test (using the dominant and non-dominant hands) is regularly used to detect the motor abnormalities in HAND [5,21], we did not include this measure because it requires equipment that is not readily available in primary care clinics. In addition, other NP tests that are significantly language-based were not included due to differences in culture. We assessed memory with the four item recall from the International HIV Dementia Scale [8]. Together, this brief battery takes between 10-15 minutes to complete.

DS (a psychiatrist trained in the use of the NP tests) trained a psychologist to administer the NP tests according to standard guidelines. In the training, the test-retest reliability between DS and the psychologist was 0.89.

### Data Analysis

To establish which sociodemographic variables were correlated with NP test performance, we analyzed multiple regression models incorporating age, gender and level of education as independent variables. In this way, we found that age was correlated with performance on DSB, the TMT-A and the TMT-B. Gender was correlated with DSB and the TMT-B. However, level of education was not significantly correlated with any of the NP test variables included here (see Table 1). Accordingly, we stratified the NP test performance according to age and gender. We present the descriptive statistics (mean, median, standard deviation) for the four NP tests and memory recall subtest (see Table 2). We divided the age into two convenient categories (30 years and >= 30 years) closest to the median. To further assess these variables we also present the skewness and kurtosis of the distributions.

**Table 1:** Results of linear multiple regression models that included age, sex and education categories in predicting NP test score outcomes.

	DSF	P	DSB	P	TMT-A	P	TMT-B	P
Age group (30-50 = 1)	-0.417^1^	0.149	-0.415	0.036	8.385	0.024	15.846	0.009

Sex (Male = 1)	-0.172	0.632	0.768	0.002	-2.093	0.646	19.041	0.012

Education group (< = 10 years = 1)	0.155	0.582	0.033	0.862	1.065	0.766	5.885	0.317

^1^Beta coefficients

DSF: digit span forward

DSB: digit span backward

TMT-A: Trail making test A

TMT-B: Trail making test B

**Table 2:** Descriptive Statistics for Age, Educational Level, Gender, Memory Item Score, Digit Span Forward, Digit Span Backward, and the Trail Making Test Parts A and memory item

	Statistics								
**Age Group 18-29 (n = 68)**	**Mean (S.D)**		**Median**	**Minimum - Maximum**		**Skewness**	**Kurtosis**	**1 SD from mean**	**2 SD from** **mean**

**Female**									

**Age**	23.29	(2.88)	23	18 -	29				

**memory**	3.77	0.47	4	2	4	-2.33	8.09	3	3

**DSF**	6.50	1.38	6	4	9	0.17	2.30	5	4

**DSB**	3.63	0.97	3	2	7	1.01	4.18	3	2

**TMT-A**	40.73	17.41	35	17	93	1.36	4.25	58	76

**TMT-B**	72.57	25.97	35.5	37	220	3.13	18.29	99	125

									

**Male**									

**Age**	22.62	(2.82)	22	19 -	29				

**memory**	3.39	0.55	3.5	2.5	4	-0.16	1.75	3	2

**DSF**	6.33	1.12	6	4	8	0.70	3.42	5	4

**DSB**	4.56	0.73	5	3	5	-1.24	3.17	4	3

**TMT-A**	35.89	8.94	35	21	55	0.67	4.05	45	54

**TMT-B**	87.78	26.51	77	61	135	0.69	2.11	115	141

**Age Group 30-50 (n = 42)**									

**Female**									

**Age**	38.39	(5.15)	39	30 -	50				

**memory**	3.66	0.48	4	2	4	-1.46	5.03	3	3

**DSF**	6.14	1.40	6	4	9	0.40	2.21	5	3

**DSB**	3.29	0.83	3	2	6	1.35	5.32	3	2

**TMT-A**	48.54	18.70	50	18	90	0.33	2.38	67	86

**TMT-B**	89.26	28.42	85	53	180	1.10	4.23	118	146

									
**Male**									

**Age**	36	(5.1)	35	29 -	43				

**memory**	3.19	0.96	3.5	1.5	4	-0.83	2.18	2	1

**DSF**	6.00	1.07	6	5	8	0.75	2.50	5	4

**DSB**	3.88	0.99	4	3	6	1.19	3.75	3	2

**TMT-A**	50.00	13.63	53	28	65	-0.43	1.76	64	77

**TMT-B**	114.25	43.10	103	67	185	0.49	1.83	157	201

Memory Item: the number of words correctly recalled out of four words.

DSF and DSB: the number of items repeated in the correct sequence.

TMT Part A and TMT Part B: the number of seconds taken to complete test.

To improve the clinical utility of the data, we present the one and two standard deviation (SD) scores of the NP tests as whole numbers. People impaired on TMT will take longer (i.e. 1 or 2 SD above the mean time taken to complete the test) and people impaired on the DSF and DSB will repeat fewer words (i.e. 1 or 2 SD below the mean number of words correctly repeated). After performing the NP tests, the scores can be referenced from Table 2 and defined from Table 3 as establishing whether a patient was in a non-HAD group (this includes asymptomatic HIV neurocognitive impairment and HIV-associated mild neurocognitive disorder) or HAD group. For example, if a participant scored 2 SD away from the mean on 2 NP tests and there was no evidence of exclusion criteria, he/she would be defined as having HAD.

**Table 3:** Diagnosis of HAD Versus Absence of HAD Based on Frascati Criteria

	Absence of HAD (incorporating MND, ANI, and No NP Impairment)	HIV-Associated dementia (HAD)
**Level of NP impairment**	No to mild NP impairment	Moderate to severe NP impairment.

**Level of Functional Status Impairment**	No to mild impairment in everyday activities; may need help with complex tasks, able to perform basic Activities of daily living, walks without assistance.	Moderate to severe impairment in everyday activities ; needs help with many or most activities

**Number SD below population norm on neuropsychological test**	1-2: ANI and MND 0-1: No NP Impairment	≥ 2.0

**Number of domains impaired**	ANI and MND: ≥ 2 HAD: ≥ 2	No NP Impairment: 0-1

**Exclusion criteria**	Absence of criteria for delirium or dementia. Condition cannot be explained by another comorbid condition e.g. substance use, infections or neuropsychiatric disorder	

## Results

**Figure 1:**
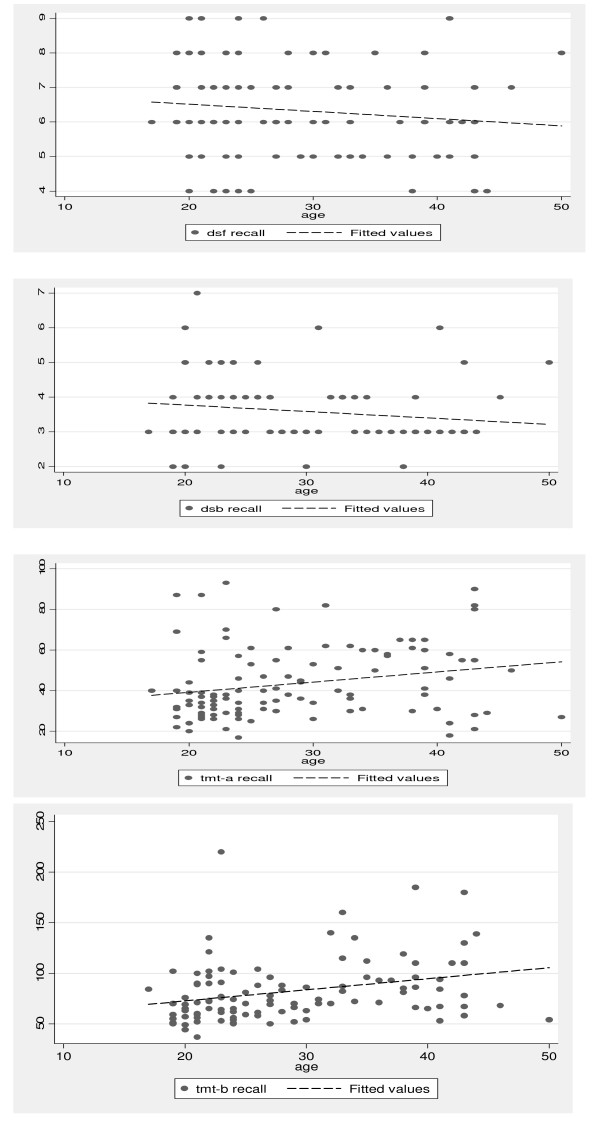
**Correlations of age and Digit Span Forward, Digit Span Backward, Trail Making Test-A and Trail Making Test-B**.

All the participants in this study were African, and they were predominantly female (84.9%). They had an average age of 28.5 (SD = 8.3) years and 10 (SD = 2) years of education. The age range was limited from 18 to 50 years. The correlation between age and the various tests are shown in Figure 1. Using tests of the line of best fit, it was demonstrated that the older adults performed worse on the NP tests. The effect of gender was not uniform across all the tests; women performed better than men on the TMT B and DSB (table 1).

There was no difference between the first and second SD of the number of items recalled for female participants; they recalled 3 items. Amongst the men, fewer items were recalled two SD from the mean. This lack of variability amongst the women will create a challenge in classifying women's performance. For example if a women scored 2 SD on one other test and recalled 3 items on the memory subtest, she can be classified as non-HAD or HAD depending if one reads 3 items in the 1 SD column or 2 SD column respectively.

## Discussion

This is the first study to recommend a brief battery of standardized NP tests and present locally relevant population norms to assess HAND in a country at the epicenter of the HIV pandemic. In particular, these tests represent a battery suitable for use by primary care physicians and allied health professionals without specialized NP training who work in busy clinical settings. Further, these NP tests assess the neurocognitive domains most frequently and earliest affected by HIV infection. Depending on the frequency and severity of abnormal NP test scores, it is possible to identify HAD in a clinical sample. The stratification of the data by age and gender allows for a more robust interpretation than the limited data published to date on other sub-Saharan African populations that provide only a summary mean and standard deviation[7].

Our findings are consistent with those of other researchers who have observed that age affects neurocognitive functioning among HIV infected persons especially on the TMT [16,22]. Several studies have assessed the relationship between the TMT and sociodemographic variables such as educational level, gender, and age. Of all the sociodemographic variables we considered, age accounted for most of the variance in NP test performance [23]. Unlike others we did not find a correlation of the TMT with educational level [24,25]. We believe this may be because our sample size was too small to detect this correlation or that years of education did not accurately reflect educational differences.

The median years of education in our sample was low (10 years) compared to other samples that had medians of 12,6 [25] and 16 years [26]. Previously available normative data are mainly from Caucasian and well-educated samples [16,21,22,24,25]. Sub-Saharan African data are highly limited. Normative data for cognitive tests has recently become available for school children in Cameroon [27]. Robertson et al [28] have provided means and standard deviations for DSF, DSB and the Color Trails Text in a Ugandan population. However, noting the confounding effect of age, educational level, and culture, the use of these summary means could lead to errors in HAND diagnosis in our population. For example, the norms on the Color Trails Test are not comparable to the time taken to complete the TMT.

Our findings support Fernandez and Marcopulos' conclusion that use of cross-cultural data for the TMT will lead to errors in classification [16]. For example, according to the norms published by Tombach (on a Caucasian population) the average time for an 18-24 year old to complete the TMT A and B is 22.93 and 48.97 seconds, respectively [25]. In our sample these same tests would require almost one and a half to twice that amount of time -- 38.92 and 70.96 seconds. The limited variability of the number of items recalled by women would limit the clinical utility of the memory subtest. For example, if a woman recalled three items it will be difficult to classify her performance because three items is the number of items on both the first and second standard deviation on this subtest (table 2). We would recommend investigators use other longer and more sensitive tests to assess verbal memory e.g., the California Verbal Learning Test II.

The strengths of this study are that we more completely present the descriptive statistical properties of NP test performance and the results of relevant stratification of data rather than the summary measures alone. These data can be used by other investigators in South Africa to standardize their own local NP test data. Given the well documented influence of age and educational level on neurocognitive performance [7,24,29], we adjusted for these co-factors. The 'normative' data thus obtained therefore may be controlled for variance according to the age and educational level of the cohort studied. Our sample was largely represented by young African females. This is the group is most frequently affected by HIV infection in sub-Saharan Africa, and, therefore, our data set serves as an appropriate reference. Our data meet many of the criteria set out by Mitrushina et al. in evaluating the norms for the TMT [24]. As recommended, we provide a detailed description of the sample, with inclusion and exclusion criteria, gender distribution, and descriptive statistical properties of the NP data obtained [24].

There are several limitations of this study. The sample size is relatively small. True population norms typically require at least a thousand people; however, this level of sampling is very costly. It may require many years before such data become available. Others have presented similarly small sample size studies [16,22,30]. The findings may not be generalizable to the entire population; however, in the absence of NP data on South Africans, the high burden of HIV disease, and the urgent need for more refined methods to diagnose HAND, this study presents data that can be used immediately by primary care clinicians and allied health professionals caring for the HIV infected. Other sub-Saharan counties can also use these data, as they may well be more comparable than other data derived on HIV infected persons from predominantly well educated, high resource, Caucasian countries.

## Conclusion

The assessment of neurocognitive status is recommended as standard evaluation for all HIV seropositive pesons. Notwithstanding the problems of current screening tests for HIV-associated dementia and the need for population norms using the new classification of HAND, this battery of NP tests does offer an alternative to other screening scales not standardized on this population and longer NP test batteries. The four NP tests we recommend to utilize (DSF, DSB, TMT A, TMT B) can be completed in 15 minutes, require minimal assessor training, and demand no specialized equipment. Primary care clinicians and allied health professionals caring for the HIV infected should be trained to use these NP tests and the available normative reference data. Data like those reported here, will assist HIV primary care providers to more reliably diagnose HAND, monitor its progression, and its response to therapy (such as CSF-penetrating ARV medications) without the need to rely upon CD4 cell count and plasma viral load to make this assessment.

## Competing interests

The authors declare that they have no competing interests.

## Authors' contributions

DS was involved in the study concept, design, data collection, data analysis and prepared the first draft of manuscript. JAJ contributed to the revision of the manuscript. LM participated in data analysis and revision of manuscript. RHP participated in interpretation of data and revision of the manuscript. SJ and HS participated in conception of study design and revision of manuscript. KG and EL made final revisions to the manuscript. All authors read and approved the final manuscript.
